# Activity Recognition for IoT Devices Using Fuzzy Spatio-Temporal Features as Environmental Sensor Fusion

**DOI:** 10.3390/s19163512

**Published:** 2019-08-11

**Authors:** Miguel Ángel López Medina, Macarena Espinilla, Cristiano Paggeti, Javier Medina Quero

**Affiliations:** 1 Council of Health for the Andalusian Health Service, Av. de la Constitución 18, 41071 Sevilla, Spain; 2 Department of Computer Science, Campus Las Lagunillas, 23071 Jaén, Spain; 3 I + Srl, Piazza G.Puccini, 26, 50144 Firenze, Italy

**Keywords:** sensor data fusion, fuzzy logic, activity recognition, smart objects

## Abstract

The IoT describes a development field where new approaches and trends are in constant change. In this scenario, new devices and sensors are offering higher precision in everyday life in an increasingly less invasive way. In this work, we propose the use of spatial-temporal features by means of fuzzy logic as a general descriptor for heterogeneous sensors. This fuzzy sensor representation is highly efficient and enables devices with low computing power to develop learning and evaluation tasks in activity recognition using light and efficient classifiers. To show the methodology’s potential in real applications, we deploy an intelligent environment where new UWB location devices, inertial objects, wearable devices, and binary sensors are connected with each other and describe daily human activities. We then apply the proposed fuzzy logic-based methodology to obtain spatial-temporal features to fuse the data from the heterogeneous sensor devices. A case study developed in the UJAmISmart Lab of the University of Jaen (Jaen, Spain) shows the encouraging performance of the methodology when recognizing the activity of an inhabitant using efficient classifiers.

## 1. Introduction

Activity Recognition (AR) defines models able to detect human actions and their goals in smart environments with the aim of providing assistance. Such methods have increasingly been adopted in smart homes [1] and healthcare applications [2] aiming both at improving the quality of care services and allowing people to stay independent in their own homes for as long as possible [3]. In this way, AR has become an open field of research where approaches based on different types of sensors have been proposed [4]. In the first stages, binary sensors were proposed as suitable devices for describing daily human activities within a smart environment setting [5,6]. More recently, wearable devices have been used to analyze activities and gestures in AR [7].

Furthermore, recent paradigms such as edge computing [8] or fog computing [9] place the the data and services within the devices where data are collected, *providing virtualized resources and engaged location-based services, at the edge of the mobile networks* [10]. In this new perspective on the Internet of Things (IoT) [11], the focus shifts from cloud computing with centralized processing [12] to collaborative networks where the smart objects *interact with each other and cooperate with their neighbors to reach common goals* [13,14]. In particular, fog computing has had a great impact, between ambient devices [15] and wearable devices [16].

In this context, the proposed work presents a methodology for activity recognition that: (i) integrates interconnected IoT devices that share environmental data and (ii) learns from the heterogeneous data from sensors using a fuzzy approach, which extracts spatial-temporal features. The outcome of this methodology is recognizing daily activities by means of an efficient and lightweight model, which can be included in the future generation of smart objects.

The remainder of the paper is structured as follows: the following subsection reviews works related to our proposal, emphasizing the main novelties we propose. Section 2 presents the proposed methodology for learning daily activities from heterogeneous sensors in a smart environment. Section 3 introduces a case study to show the utility and applicability of the proposed model for AR in the smart environment of the University of Jaen. Finally, in Section 4, conclusions and ongoing works are discussed.

### 1.1. Related Works

Connectivity plays an important role in Internet of Things (IoT) solutions [17]. Fog computing approaches require the real-time distribution of collaborative information and knowledge [18] to provide a scalable approach in which the heterogeneous sensors are distributed to dynamic subscribers in real time. In this contribution, smart objects are defined as both sources and targets of information using a publish-subscribe model [19]. In the proposed methodology, we define a fog computing approach to: (i) distribute and aggregate information from sensors, which are defined by spatial-temporal features with fuzzy logic, using middleware based on the publish-subscribe model, and (ii) learn from the distributed feature sensors with efficient classifiers, which enable AR within IoT devices.

In turn, in the context of intelligent environments, a new generation of non-invasive devices is combined with the use of traditional sensors. For example, the use of new location devices based on UWB is allowing us to reach extremely high accuracy in indoor contexts [20], which has increased the performance of previous indoor positioning systems based on BLE devices [21]. At the same time, the use of inertial sensors in wearable devices has been demonstrated to enhance activity recognition [22]. These devices coexist with traditional binary sensors, which have been widely used to describe daily user activities from initial AR works [23] to more recent literature [24]. These heterogeneous sensors require integrating several sources: wearable, binary, and location sensors in smart environments [25], to enable rich AR by means of sensor fusion [26].

Traditionally, the features used to describe sensors under data-driven approaches have depended on the type of sensors, whether accelerometers [27] or binary sensors [28]. In previous works, deep learning has also been shown as a suitable approach in AR to describe heterogeneous features from sensors in smart environments [5,6,29]. However, it is proving hard to include learning capabilities in miniature boards or mobile devices integrated in smart objects [30]. First, we note that deep learning requires huge amounts of data [31]. Second, learning, and in some cases, evaluating, under deep learning approaches within low computing boards requires the adaptation of models and the use of costly high-performance embedded boards. In line with this, we highlight the work [32], where a new form of compression models was proposed in several areas to deploy deep neural networks in high-performance embedded processors, such as the ARM Cortex M3. Advancement across a range of interdependent areas, including hardware, systems, and learning algorithms, is still necessary [33].

To bring the capabilities needed to develop general features from sensors to low-computing devices, we propose using spatial-temporal feature extraction based on fuzzy logic with minimal human configuration together with light and efficient classifiers. On the one hand, fuzzy logic has been proposed in sensor fusion [34] and the aggregation of heterogeneous data in distributed architectures [35]. For example, fuzzy temporal windows have increased performance in several datasets [5,6,29], extracting several temporal features from sensors, which have been demonstrated as a suitable representation for AR from binary [36] and wearable [37] sensors.

On the other hand, some other efficient classifiers have been successfully proposed for AR [38] using devices with limited computing power. For example, decision trees, k-nearest neighbor, or support vector machine has enabled AR in ubiquitous devices by processing embedded sensors in mobile devices [39,40].

Taking this research background into consideration, we defined the following key points to include in our approach and compensate for the lack of previous models:

- To share and distribute data from environmental, wearable, binary, and location sensors among each other using open-source middleware based on MQTT [17].
- To extract spatial features from sensors using fuzzy logic by means of fuzzy scales [41] with multi-granular modeling [42].
- To extract temporal features in the short- and middle-term using incremental fuzzy temporal windows [5].
- To learn from a small amount of data, to avoid the dependency of deep learning on a large amount of data [31].
- To evaluate the performance of AR with efficient and lightweight classifiers [40], which are compatible with computing in miniature boards [38].

## 2. Methodology

In this section, we present the proposed methodology for learning daily activities from heterogeneous sensors in a smart environment. As the main aim of this work is integrating and learning the information from sensors in real time, we first describe them formally. A given sensor $s^{i}$ provides information from a data stream $S^{i}\left(t^{*}\right)=\left\{v_{t^{0}}^{i},v_{t^{1}}^{i},\ldots ,v_{t^{N}}^{i}\right\}$, where $v_{t}^{i}$ represents a measurement of the sensor $s^{i}$ in the timestamp *t*. Under real-time conditions, $t^{*}$ represents the current time and $S^{i}\left(t^{*}\right),t^{*}\geq t$ the status of the data stream in this point in time.

In this work, in order to increase scalability and modularity in the deployment of sensors, each sensor $s^{i}$ publishes the data stream independently of the other sensors in real time. For this, a collecting rate Δt is defined in order to describe the data stream constantly and symmetrically over time:

$$S^{i}\left(t^{*}\right)=\left\{v_{t^{*}}^{i},v_{t^{*}-\Delta t}^{i},\ldots ,v_{t^{*}-\Delta t\cdot j}^{i}\right\}$$

Further details on the deployment of sensors in real time are presented in Section 2.1, where a new trend for smart objects and devices are connected with each other using publish-subscribe-based middleware.

Next, in order to relate the data stream with the activities performed by the inhabitant, it is necessary to describe the information from the sensor stream with a set of features $F=\{F_{1},\ldots ,F_{\left|F\right|}\}$, where a given feature $F_{m}$ is defined by the function $F_{m}\left(S^{i},t^{*}\right)$ to aggregate the values $v_{t_{j}}^{i}$ of the sensor streams $S^{i}$ in the current time $t^{*}$:

$$F_{k}\left(S^{i},t^{*}\right)=\overset{t^{*}>t_{j}}{\underset{t_{j}}{⋃}}v_{t_{j}}^{i}$$

Since our model is based on a data-driven supervised approach, the features that describe the sensors are related to a given label $L(t^{*})$ for each given time $t_{*}$:

$$F_{1}\left(S^{1},t^{*}\right),\ldots ,F_{m}\left(S^{i},t^{*}\right),\ldots ,F_{|}F|\left(S^{N},t^{*}\right)\to L\left(t^{*}\right)$$

where $L(t^{*})$ defines a discrete value $L=\{L_{1},\ldots ,L_{l}\ldots ,L_{\left|L\right|}\}$ and $L_{l}$ identifies the labeled activity performed by the inhabitant in the given time $t_{*}$.

In Section 2.2, we describe a formal methodology based on fuzzy logic to obtain spatial-temporal features to fuse the data from the heterogeneous sensors.

Next, we describe the technical and methodological aspects.

### 2.1. Technical Approach

In this section, we present sensors and smart objects that have been recently proposed as non-invasive data sources for describing daily human activities, followed by the middleware used to interconnect these different devices.

#### 2.1.1. Smart Object and Devices

As mentioned in Section 1.1, the aim of this work is to enable the interaction between new generations of smart objects. In this section, we present the use of sensors and smart objects, which have been recently proposed as non-invasive data sources for describing daily human activities. These devices have been included in the case study presented in this work, deployed at the the UJAmISmart Lab of University of Jaen (Jaen, Spain) [25] (http://ceatic.ujaen.es/ujami/en/smartlab).

**Figure 1:**
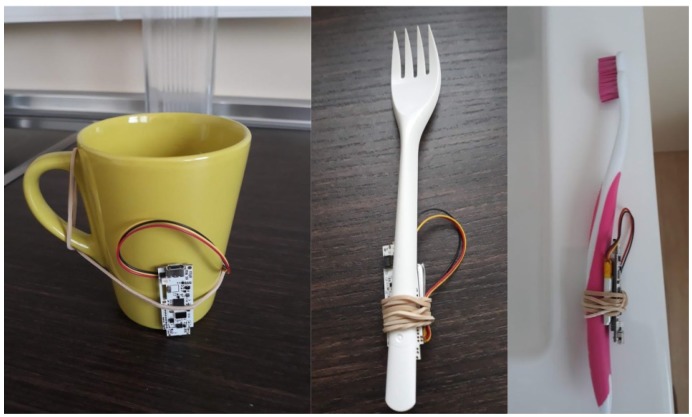
Prototyping of smart objects (a cup, a toothbrush, and a fork), whose orientation and movement data are collected and sent in real time by an inertial miniature board (The Tactigon).

First, in order to gain data on certain objects for AR, we included an inertial miniature board in some daily-use objects, which describes their movement and orientation. To evaluate this information, we attached a Tactigon board [43] to them, which collects inertial data from the accelerometer and sends them in real time under a BLE protocol. For prototyping purposes, in Figure 1, we show the integration of the inertial miniature board in some objects.

Second, we acquired indoor location data by means of UWB devices, which offer high performance with a location accuracy closer to centimeters [44], using wearable devices carried by the inhabitants of the smart environment [44]. In this work, we integrated Decawave’s EVK1000 device [45], which is configured with (almost three) anchors located in the environment and one tag for each inhabitant.

Third, as combining inertial sensors from wearable devices on the user enhances activity recognition [22], we collected inertial information from a wristband device worn by an inhabitant. In this case, we developed an Android Wear application deployed in a Polar M600, which runs on Android Wear [46]. The application allowed us to send data from the accelerometer sensor in real time through a WiFi connection.

Fourth, we included binary sensors in some static objects that the inhabitant interacts with while performing his/her daily activities, such as the microwave or the bed. For this purpose, we integrated some smart things devices [47] in the UJAmI Smart Lab, which transmit the activation of pressure from a mat or the open-close of a door through a Z-Wave protocol. These four types of sensors represent a new trend of high-capability devices for AR. In the next section, we describe the architecture to connect these heterogeneous sources and distribute sensor data in real time.

#### 2.1.2. Middleware for Connecting Heterogeneous Devices

In this section, we describe a distributed architecture for IoT devices using MQTT, where aggregated data from sensors are shared under the publish-subscribe model. The development of an architecture to collect and distribute information from heterogeneous sensors in smart environments has become a key aspect, as well as the prolific research field in the integration of IoT devices due to the lack of standardization [48]. In this section, we present the middleware deployed at the UJAmI Smart Lab of the University of Jaen (Spain) [25] based on the following points:

- We include connectivity for devices in a transparent way, including BLE, TCP, and Z-Wave protocols.
- The data collected from heterogeneous devices (without WiFi capabilities) are sent to a given gateway, which reads the raw data in a given protocol, aggregates them, and sends them by MQTT under TCP.
- The representation of data includes the timestamp for when the data were collected together with the given value of the sensor. The messages in MQTT describe the data in JSON format, a lightweight, text-based, language-independent data interchange format [49].

Next, we describe the specific configuration for each sensor deployed in this work. First, the inertial data from the miniature boards located in smart objects are sent under BLE in raw format at a frequency close to 100 samples per second. A Raspberry Pi is configured as a BLE gateway, reading information from the Tactigon boards, aggregating the inertial data into one-second batches and sending a JSON message in MQTT on a given topic for each sensor.

Second, the Decawave UWB devices are collected in a gateway at a frequency close to 1 sample per second, collecting the location of tag devices by means of a USB connection. The open-source software (https://www.decawave.com/software/) from Decawave was used to read the information and then publish a JSON message in MQTT with the location on a given topic for each tag.

Third, an Android Wear application was developed in order to collect the information from the inertial sensors of the smart wristband devices. The application obtains acceleration samples at a frequency close to 100 per second, collecting a batch of aggregated samples and publishing a JSON message in MQTT on a given topic for each wearable device.

Fourth, a Raspberry Pi is configured as a Z-Wave gateway using a Z-Wave card connected to the GPIO and the software (https://z-wave.me/). In this way, the Raspberry Pi gateway is connected to smart things devices in real time, receiving the raw data and translating them to JSON format to be published in real time using MQTT on a given topic that identifies the device.

**Figure 2:**
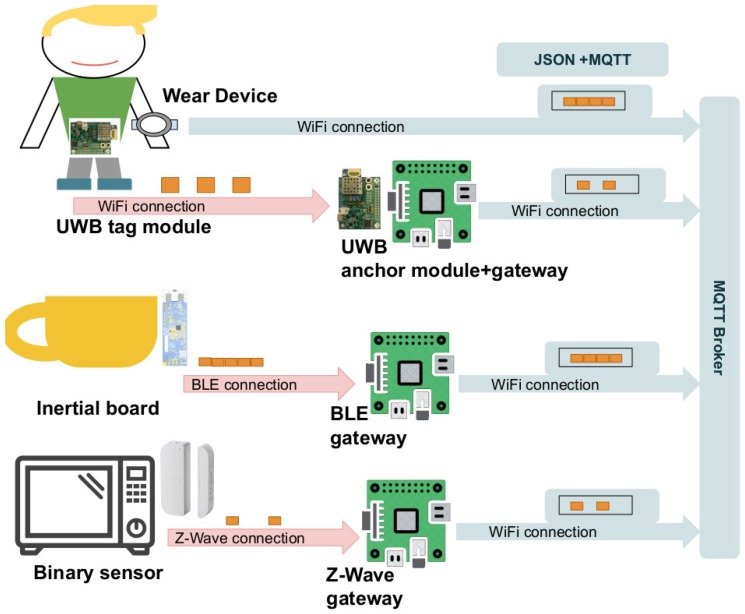
Architecture for connecting heterogeneous devices. Binary, location, and inertial board sensors send raw data to gateways, which collect, aggregate, and publish data with MQTT in JSON format. The Android Wear application collects, aggregates, and publishes the data directly using MQTT through WiFi connection.

In Figure 2, we show the architecture of the hardware devices and software components that configure the middleware for distributing the heterogeneous data from sensors in real time with MQTT in JSON format.

### 2.2. Fuzzy Fusion of Sensor Spatial-Temporal Features

After detailing the technical configuration of the devices and middleware involved in this work, we present a methodology used to extract spatial-temporal features and represent the heterogeneous data from sensors using fuzzy logic in a homogeneous way in order to learn and evaluate tasks in activity recognition using light and efficient classifiers.

The proposed methodology is based on the following stages:

- Describing the spatial representation of sensors by means of fuzzy linguistic scales, which provide high interpretability and require minimal expert knowledge, by means of ordered terms.
- Aggregating and describing the temporal evolution of the terms from linguistic scales by means of fuzzy temporal windows including a middle-to-short temporal evaluation.
- Predicting AR from the fused sensor features by means of light classifiers, which can be trained and evaluated in devices with low computing power.

**Figure 3:**
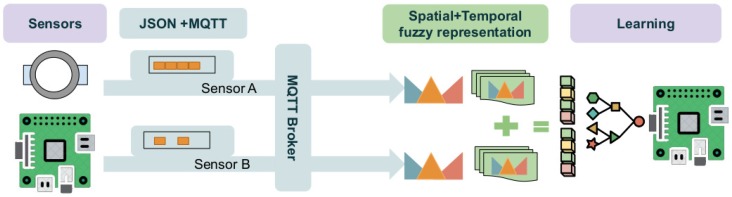
Fuzzy fusion of spatial-temporal features of sensors: (i) data from the heterogeneous sensors are distributed in real time; (ii) fuzzy logic processes spatial-temporal features; (iii) a light and efficient classifier learns activities from the features.

In Figure 3, we show the components involved in fusing the spatial-temporal features of heterogeneous sensors.

#### 2.2.1. Spatial Features with Fuzzy Scales

In this section, we detail how the data from heterogeneous sensors is described using fuzzy scales, requiring minimal expert knowledge. A fuzzy scale $\bar{|L^{i}|}$ of granularity *g* describes the values of an environmental sensor $s^{i}$, which is defined by the terms $A_{l}^{i},l\in \left[1,g\right]$. The terms within the fuzzy linguistic scale (i) fit naturally and are equally ordered within the domain of discourse of the sensor data stream $S^{i}$ from the interval values $\left[L_{1},\ldots \;L_{g}\right]$ and (ii) fulfill the principle of overlapping to ensure a smooth transition [50].

(1) $$\begin{array}{c} \bar{|L^{i}|}=\left\{\bar{A_{1}^{i}},\ldots ,\bar{A_{l}^{i}},\ldots ,\bar{A_{g}^{i}}\right\}, \end{array}$$

Each term $A_{l}^{i},l\in \left[1,g\right]$ is characterized by using a triangular membership function as detailed in Appendix A. Therefore, the terms $A_{l}^{i}$, which describe the sensor $s^{i}$, configure the fuzzy spatial features of the sensor from the values:

$$A_{l}^{i}\left(t^{*}\right)=A_{l}^{i}\left(S^{i}\left(t^{*}\right)\right)=\left\{A_{l,t^{*}}^{i}\left(v_{t^{*}}^{i}\right),\ldots ,A_{l,j}^{i}\left(v_{j}^{i}\right)\right\}$$

**Figure 4:**
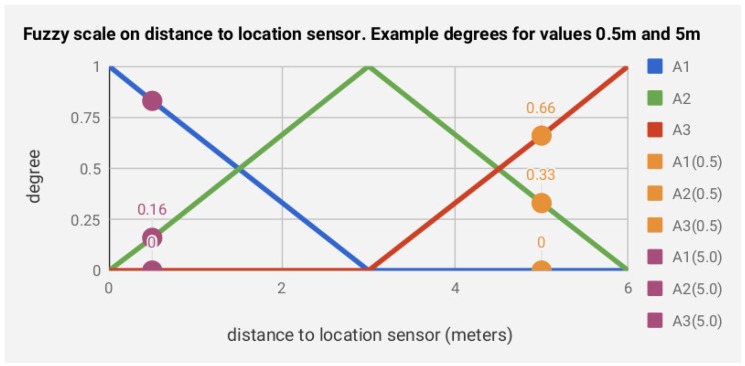
Example of the fuzzy scale defined for g=3 on distance to the location sensor. Example degrees for values evaluating the distances $S^{i}\left(t^{*}\right)=\left\{v_{t_{1}}=0.5\;m,v_{t_{2}}=5.0\;m\right\}\to A_{1}\left(t^{*}\right)=\left\{A_{1,t_{1}}=0,A_{1,t_{2}}=0.83\right\},A_{2}\left(t^{*}\right)=\left\{A_{2,t_{1}}=0.33,A_{2,t_{2}}=0.16\right\},A_{3}\left(t^{*}\right)=\left\{A_{3,t_{1}}=0.66,A_{3,t_{2}}=0\right\}$.

To show a graphical description of the use of fuzzy linguistic scales in describing the sensor values, we provide an example of the sensor location in Figure 4. In the example, the location sensor *s* measured the distance in meters to the inhabitant within a maximum of 6 meters (we avoided the sensor super index for the sake of simplicity), whose sensor stream $S^{i}\left(t^{*}\right)=\left\{v_{t_{1}}=0.5\;m,v_{t_{2}}=5.0\;m\right\}$ is defined in the two points of time $t_{1}$ and $t_{2}$. First, we describe a fuzzy scale $\bar{\left|L\right|}$ of granularity g=3, which determines the membership function of the terms $A_{1},A_{2},A_{3}$. Second, from the values of the sensor stream, which define the distances to the location sensor, we computed the degrees for each term $A_{1},A_{2},A_{3}$ in the fuzzy scale obtaining $A_{1}\left(t^{*}\right)=\left\{A_{1,t_{1}}=0,A_{1,t_{2}}=0.83\right\},A_{2}\left(t^{*}\right)=\left\{A_{2,t_{1}}=0.33,A_{2,t_{2}}=0.16\right\},A_{3}\left(t^{*}\right)=\left\{A_{3,t_{1}}=0.66,A_{3,t_{2}}=0\right\}$.

#### 2.2.2. Temporal Features with Fuzzy Temporal Windows

In this section, the use of multiple Fuzzy Temporal Windows (FTW) and fuzzy aggregation methods [35] is proposed to enable the short- and middle-term representation [5,6] of the temporal evolution of the degrees for the terms $A_{l}^{i}\left(t^{*}\right)$.

The FTWs are described straightforwardly according to the distance from the current time $t^{*}$ to a given timestamp $t^{j}$ as $\Delta t^{j}=t^{*}-t^{j}$ using the membership function $\mu_{T_{K}\left(\Delta t^{j}\right)}$. Therefore, a given FTW $T_{k}$ is defined by the values $L_{k},L_{k-1},L_{k-2},L_{k-3}$, which determine a trapezoidal membership function (referred to in Appendix C), as:

(2) $$T_{k}=T_{k}\left(\Delta t_{i}^{*}\right)\left[L_{k},L_{k-1},L_{k-2},L_{k-3}\right]$$

Next, the aggregation degree from the relevant terms $A_{l}^{i}\left(t_{j}\right)$ within the temporal window $T_{K}^{i}$ of a sensor $s^{i}$ is computed using a max-min operator [35] (detailed in Appendix B). This aggregation degree is defined as $A_{l}^{i}\left(t^{*}\right)\cup T_{k}^{i}\left(t^{*}\right)$, which represents the aggregation degree of the FTW $T_{K}^{i}$ over the degrees of term $A_{l}^{i}\left(t^{*}\right)$ in a given time $t^{*}$.

**Figure 5:**
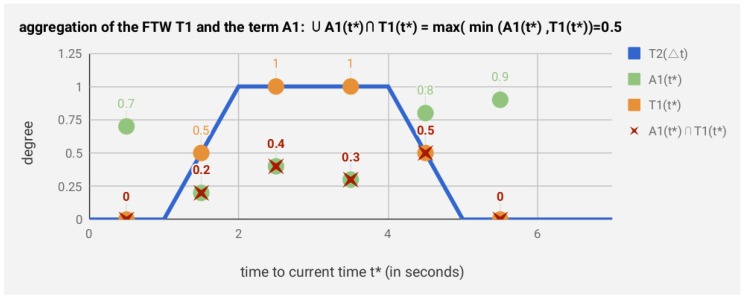
Example of temporal aggregation of the FTW $T_{1}\left(\Delta t_{i}^{*}\right)$[1 s, 2 s, 4 s, 5 s] (in magnitude of seconds *s*) for the degrees of the term $A_{1}\left(t^{*}\right)=\left\{0.7,0.2,0.4,0.3,0.5,0.9\right\}$. The aggregation degree $A_{1}\left(t^{*}\right)\cup T_{1}\left(t^{*}\right)=0.5$ is determined by the max-min operator. The value 0.5 defines a fuzzy spatial temporal feature of the sensor stream.

We provide an example in Figure 5 to show a graphical description of the use of an FTW $T_{1}\left(\Delta t^{j}\right)$ in aggregating the degrees of a term $A_{1}$ in the sensor stream as $A_{1}\left(t^{*}\right)=\left\{A_{1,t_{1}}=0.7,A_{1,t_{2}}=0.2,A_{1,t_{3}}=0.4,A_{1,t_{4}}=0.3,A_{1,t_{5}}=0.5,A_{1,t_{6}}=0.9\right\}$ (we avoid the sensor super index for the sake of simplicity). First, we define an FTW as $T_{1}=T_{1}\left(\Delta t_{i}^{*}\right)$[1 s, 2 s, 4 s, 5 s] $\bar{\left|L\right|}$ in magnitude of seconds *s*. Second, we compute the degree of the temporal window $T_{1}\left(t^{*}\right)=\{t_{1}=0,t_{2}=0.5,t_{3}=1,t_{4}=1,t_{5}=0.5,t_{6}=0$, whose aggregation degree $A_{1}\left(t^{*}\right)\cup T_{1}\left(t^{*}\right)$ is computed by the max-min operator and determines the value of the spatial-temporal feature defined by the pair $T_{1},A_{1}$. Therefore, we define a given feature $F_{m}=A_{l}^{i}\left(t^{*}\right)\cup T_{k}^{i}\left(t^{*}\right)$ for each pair of fuzzy terms $A_{l}^{i}$ and the FTW $T_{K}^{i}$ of a sensor stream $S^{i}$ in the current time $t^{*}$.

## 3. Results

In this section, we describe the experimental setup and results of a case study developed at the UJAmI Smart Lab of the University of Jaen (Spain), which were gathered in order to evaluate the proposed methodology for AR.

### 3.1. Experimental Setup

The devices defined in Section 2.1.1 were previously deployed at the UJAmI Smart Lab of the University of Jaen. The middleware based on MQTT and JSON messages integrated: (i) UWB-Decawave location devices, (ii) Tactigon inertial devices, (iii) Smart Things binary sensors, (iv) wearable devices (Polar M660) with Android Wear, and (v) Raspberry Pi gateways. The middleware allowed us to collect data from environmental sensors in real time: location and acceleration data from inhabitants; acceleration data from three smart objects: a cup, a toothbrush, and a fork; binary activation from nine static objects: bathroom faucet, toilet flush, bed, kitchen faucet, microwave, TV, phone, closet, and main door.

In the case study, 5 scenes were collected while the inhabitant performed 10 activities: sleep, toileting, prepare lunch, eat, watch TV, phone, dressing, toothbrush, drink water and enter-leave house. A scene consisted of a coherent sequence of human actions in daily life, such as: waking up, preparing breakfast, and getting dressed to leave home. In the 5 scenes, a total of 842 samples for each one of the 26 sensors were recorded in one-second time-steps. Due to the high inflow of data from the inertial sensors, which were configured to 50 Hz, we aggregated the data in one-second batches within the gateways. Other sensors sent the last single value for each one-second step from the gateways where they were connected. In Table 1, we provide a description of the case scenes and in Table 2 the frequency of activities.

**Figure 6:**
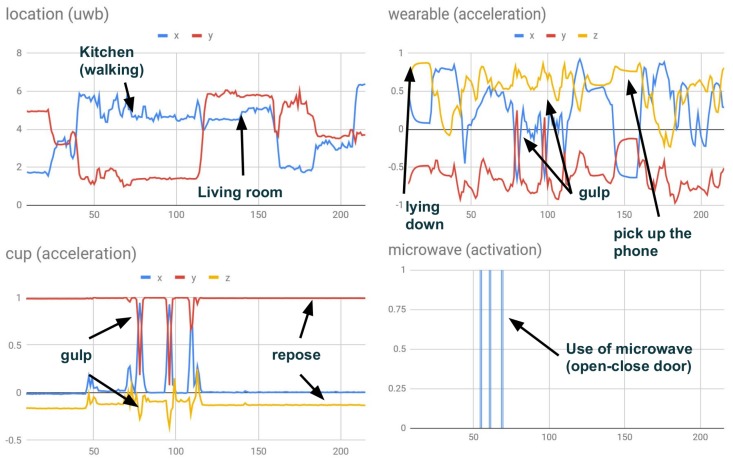
Data from heterogeneous sensors. The top-left shows the location in meters from a UWB device. The top-right shows acceleration from a wearable device. The bottom-left shows acceleration in the inhabitant’s cup. The bottom-right shows the activation of the microwave. Some inhabitant behaviors and the impact on sensors are indicated in the timelines.

The information from all sensors was distributed in real time by means of MQTT messages and topics in one-second time-steps. An MQTT subscriber collected and recorded the sensor data from topics streaming within a database. The collection of data was managed by MQTT messaging, enabling us to start or stop data collection in the database in real time. We note that at the same point of time, each board or computer could take different time-stamps since the clocks did not have to be synchronized. To synchronize all the devices (within the one-second interval), we collected the time-stamp of the first value for each sensor from the initial message for collecting data, which determined the reference time $t_{0}$ for this sensor. Therefore, all the following timestamps for each sensor were computed as relative time to starting time $t_{i}^{\prime }=t_{i}-t_{0}$. Some examples of data collected from different sources are shown in Figure 6.

During the case study, an external observer labeled the timeline with the activity carried out by the inhabitant in real time. For training and evaluation purposes, a cross-validation was carried out with the 5 scenes (each one was used for testing and another for training). The evaluation of the AR was developed in streaming for each second in real-time conditions, without explicit segmentation of the activities performed. Next, we merged all time-steps from the 5 case tests, configuring a full timeline test, which could be analyzed according to the metrics. The metrics to evaluate the models were precision, recall, and F1-score, which were computed for each activity. In turn, we allowed an error margin of a second, since the human labeling of the scenes may be slightly displaced at this speed (by a margin of seconds).

Finally, as light and efficient classifiers, we evaluated: kNN, decision tree (C4.5), and SVM, whose implementation in Java and C++ [51,52,53] enable learning and evaluation capabilities in miniature boards or mobile devices. We evaluated the approach in a mid-range mobile device (Samsung galaxy J7), where the classifiers were integrated using Weka [52] and the learning time of the classifier was measured.

### 3.2. Baseline Features

**Figure 7:**
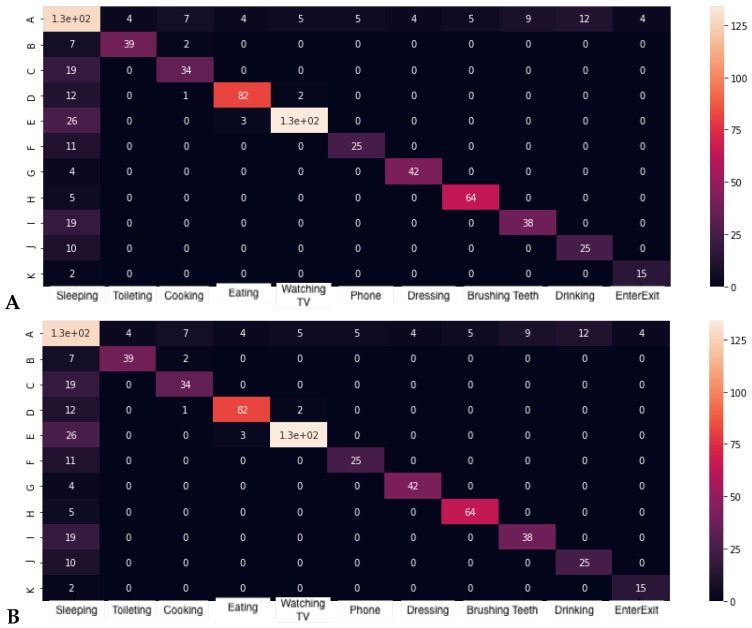

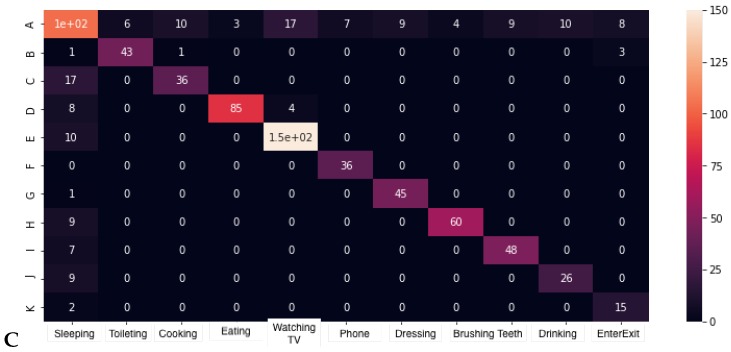
Confusion matrix for the best classifiers. (**A**) SVM + $\left[t^{+},t^{-}\right]=\left[0,1\right]$ in the baseline, (**B**) KNN + $\left[t^{+},t^{-}\right]=\left[2,4\right]$ with fuzzy spatial features, and (**C**) KNN + $\left[t^{+},t^{-}\right]=\left[8,15\right]$ with fuzzy spatial temporal features.

In this section, we present the results of baseline features in AR using raw data provided by sensors. Therefore, first we applied the classification of raw data collected by middleware for each second and activity label. Second, in order to evaluate the impact of aggregating raw data in a temporal range, we included an evaluation of several sizes of temporal windows, which summarized sensor data using maximal aggregation. The configurations were: (i) $\left[t^{+},t^{-}\right]=\left[0,1\right]$, (ii) $\left[t^{+},t^{-}\right]=\left[1,3\right]$, and (iii) $\left[t^{+},t^{-}\right]=\left[2,5\right]$, where $\left[t^{+},t^{-}\right]$ configure the given temporal window $\left[t^{*}+t^{+},t^{*}-t^{-}\right]$ for each evaluation time $t^{*}$ in the timeline. The number of features corresponds to the number of sensors |S|. Results and learning time in mobile devices for each activity and classifier are shown in Table 3; the confusion matrix with the best configuration $\left[t^{+},t^{-}\right]=\left[0,1\right]$ and SVM is shown in Figure 7.

We can observe that the use of one temporal window with baseline features was only suitable when the window size fit the short-term sensor activation $\left[t^{+},t^{-}\right]=\left[0,1\right]$.

### 3.3. Fuzzy Spatial-Temporal Features

In this section, we detail the extraction of fuzzy spatial-temporal features from the sensors of the case study. First, in order to process the data from the UWB and acceleration sensors (in wearable devices and inertial objects), we applied a normalized linguistic scale $L^{i}$ with granularity g=3, where the proposed linguistic terms fit naturally ordered within the domain of discourse of the environmental sensor.

The number of features correspond to the number of sensors times granularity |S|×g. The linguistic scale of UWB location was defined in the domain [0,6] m since the the smart lab is less than six meters in size, and the linguistic scale of acceleration data was between the normalized angles defined in [−1,1]. Binary sensors which are represented by the values 0,1, one in the case of activation, have the same straightforward representation as a fuzzy or crisp value.

In Table 4, we present the results and learning time in a mobile device for each activity and classifier; the confusion matrix with the best configuration $\left[t^{+},t^{-}\right]=\left[2,5\right]$ and kNN are shown in Figure 7. We note the stability of the results in the different windows compared to the previous results without fuzzy processing.

Second, we applied two configurations of FTWs to represent middle- and short-term activation of sensors: (i) $\left[t^{+},t^{-}\right]=\left[3,5\right]\to T_{1}^{-}=\left\{-5,-5,-3,-2\right\},T_{0}=\left\{-3,-2,2,3\right\},T_{1}^{+}=\left\{0,0,2,3\right\}$ and (ii) $\left[t^{+},t^{-}\right]=\left[8,13\right]\to T_{1}^{-}=\left\{-13,-13,-3,-2\right\},T_{2}=\left\{-3,-2,2,3\right\},T_{1}^{+}=\left\{0,0,3,8\right\}$, where $T_{1}$ represents a past fuzzy temporal window, $T_{2}$ a fuzzy temporal window closer to current time $t^{*}$, and $T_{3}$ a delayed temporal window from the current time $t^{*}$. The first and second configurations contained a further temporal evaluation of 8 s and 21 s, respectively. The number of features corresponds to the number of sensors times granularity and the number of temporal windows |S|×g×|T|.

Finally, in Table 5, we present the results and learning time in a mobile device for each activity and classifier; the kNN confusion matrix is shown in Figure 7. We note the increase of performance when including several fuzzy temporal windows highlighting the learning time, efficiency, and f1-score of kNN.

### 3.4. Representation with Extended Baseline Features

In this section, we evaluate the impact of including an advanced representation of sensors as baseline features. For inertial sensors, we included the aggregation function: maximal, minimal, average, and standard deviation, which are identified as a strong representation of acceleration data [27]. In the case of binary sensors, we included the last activation of the sensors and the current activation to represent the last status of the smart environment, which has brought about encouraging results in activity recognition [28,54]. These new features were computed to obtain an extended sensor representation.

First, we computed the performance of the extended representation when used as baseline features within one-second windows $\left[t^{+},t^{-}\right]=\left[0,1\right]$, which can be compared with Table 3 to see how the results correspond with the non-extended features. Second, we evaluated the impact of applying the fuzzy spatial-temporal methodology to the extended features with the configurations for FTW $\left[t^{+},t^{-}\right]=\left[8,13\right]$ and $\left[t^{+},t^{-}\right]=\left[3,5\right]$, which can be compared with Table 5 to see how the results correspond with the non-extended features. The results with the performance of the extended representation are shown in Table 6.

### 3.5. Impact on Selection by Type of Sensor

In this section, we evaluate the impact of selecting a subset of the sensors of the case study on activity recognition. For this, we started with the best configuration, which utilized fuzzy spatial-temporal extended features with FTW $\left[t^{+},t^{-}\right]=\left[8,13\right]$. From this configuration, we evaluated four subsets of sensors by type:

- (S1) removing binary (using inertial and location) sensors.
- (S2) removing location (using binary and inertial) sensors.
- (S3) removing inertial (using binary and location) sensors.
- (S4) removing binary and location (using only inertial) sensors.

In Table 7, we show the results of selecting the four subsets of sensors by type.

### 3.6. Discussion

Based on the results shown in the case study, we defend that the use of fuzzy logic to extract spatial-temporal features from heterogeneous sensors constitutes a suitable model for representation and learning purposes in AR. First, spatial representation based on fuzzy scales increased performance regarding crisp-raw values. Second, the impact of including multiple fuzzy temporal windows as features, which enables middle- and short-term representation of sensor data, brought about a relevant increase in performance. Moreover, two configurations of FTWs were evaluated showing similar results, suggesting that window size definition is not critical in modeling FTW parameters, unlike with crisp windows and baseline features. Third, fuzzy spatial-temporal features showed encouraging performance from raw sensor data; however, we evaluated an advanced representation for inertial sensors and binary sensors. The use of extended features increased performance slightly by around 1–2%. Fourth, we evaluated the impact of removing some types of sensors in the deployment of the smart lab. The combination of all types of sensors provided the best configuration, and we note: (i) the use of inertial sensors and smart objects only by the inhabitant reduced performance notably; (ii) the combination of binary sensors with location or inertial sensors was closer to the best approach, which featured all of them. Finally, it is noteworthy that kNN showed encouraging results, together with SVM. The shorter learning time and the high f1-sc of kNN in AR suggest that it is the best option as a classifier to be integrated in learning AR within miniature boards. Decision trees had lower performance due to poor capabilities in analyzing continuous data.

## 4. Conclusions and Ongoing Works

The aim of this work was to describe and fuse the information from heterogeneous sensors in an efficient and lightweight manner in order to enable IoT devices to compute spatial-temporal features in AR, which can be deployed in fog computing architectures. On the one hand, a case study with a combination of location, inertial, and binary sensors was performed in a smart lab where an inhabitant carried out 10 daily activities. We included the integration of inertial sensors in daily objects and high-precision location sensors as novel aspects using middleware based on MQTT. On the other hand, we showed the capabilities of fuzzy scales and fuzzy temporal windows to increase the spatial-temporal representation of sensors. We highlighted that the results showed stable performance with fuzzy temporal windows, which helped with the window size selection problem. On spatial features, we applied the same general method based on linguistic scales to fuse and describe heterogeneous sensors. We evaluated the impact of removing the sensors by type (binary, location, and inertial), which provided relevant feedback on which ones performed better for activity recognition in a smart lab setting. Finally, we note the high representativeness of fuzzy logic in describing features, which was made the most of by the use of straightforward and efficient classifiers, among which the performance of kNN stood out.

## Figures and Tables

**Table 1:** Sequence activities of the case scenes.

Scene 1	Sleep → Toilet → Prepare lunch → Eat → Watch TV → Phone → Dressing → Toothbrush → Exit
Scene 2	Enter → Drinking → Toilet → Phone → Exit
Scene 3	Enter → Drinking → Toilet → Dressing → Cooking → Eat → Sleep
Scene 4	Enter → Toilet → Dressing → Watching TV → Cooking → Eat → Toothbrush → Sleep
Scene 5	Enter → Drinking → Toilet → Dressing → Cooking → Eat → Phone → Toothbrush → Sleep

**Table 2:** Frequency (number of time-steps) for each activity and scene.

Activity	Scene 1	Scene 2	Scene 3	Scene 4	Scene 5
Sleep	10	0	16	11	11
Toilet	13	10	6	10	14
Cooking	29	0	27	17	24
Eat	36	0	40	46	41
Watch TV	20	0	0	16	0
Phone	14	17	0	0	15
Dressing	15	0	21	16	17
Toothbrush	21	0	0	18	18
Exit	3	2	0	0	0
Enter	0	3	4	2	3
Drinking	0	16	7	0	12

**Table 3:** Results with baseline features: precision (Pre), recall (Rec) and F1-score (F1-sc).

$\left[t^{+},t^{-}\right]=\left[0,1\right]$	**SVM**			**kNN**			**C4.5**		
**Activity**	**Pre**	**Acc**	**F1-sc**	**Pre**	**Acc**	**F1-sc**	**Pre**	**Acc**	**F1-sc**
Sleep	94.73	77.08	85.00	94.73	77.08	85.00	65.45	83.33	73.31
Toilet	95.83	54.71	69.66	68.75	88.67	77.45	86.27	90.56	88.36
Cooking	66.38	89.69	76.29	58.02	75.25	65.52	50.48	67.01	57.08
Eating	81.43	92.02	86.40	80.36	93.86	86.59	76.97	77.30	77.13
Watching TV	100	94.44	97.14	94.73	94.44	94.59	91.17	91.66	91.42
Phone	100	93.47	96.62	100	100	100	97.22	95.65	96.43
Dressing	90.00	94.20	92.05	93.24	97.10	95.13	88.00	76.81	82.02
Brushing Teeth	97.36	73.68	83.88	76.27	89.47	82.34	70.96	50.87	59.26
Drinking	56.25	45.71	50.43	32.25	57.14	41.23	32.85	80.00	46.58
Enter/Exit	100	88.23	93.75	78.94	94.11	85.86	86.66	94.11	90.23
**Average**	**88.20**	**80.32**	**83.12**	**77.73**	**86.71**	**81.37**	**74.60**	**80.73**	76.23
$\left[t^{+},t^{-}\right]=\left[1,3\right]$	**SVM**			**kNN**			**C4.5**		
**Activity**	**Pre**	**Acc**	**F1-sc**	**Pre**	**Acc**	**F1-sc**	**Pre**	**Acc**	**F1-sc**
Sleep	90.47	77.08	83.24	90.47	77.08	83.24	75.00	56.25	64.28
Toilet	66.67	64.15	65.38	72.13	88.67	79.55	64.06	83.01	72.31
Cooking	80.95	87.62	84.15	63.88	61.85	62.85	63.63	56.70	59.96
Eating	84.65	93.86	89.02	82.53	92.02	87.01	88.28	71.16	78.80
Watching TV	94.87	97.22	96.03	94.87	97.22	96.03	47.05	36.11	40.86
Phone	94.44	100	97.14	97.72	93.47	95.55	97.82	93.47	95.60
Dressing	94.73	100	97.29	90.36	100	94.93	84.61	68.11	75.47
Brushing Teeth	82.22	68.42	74.68	73.91	71.92	72.90	93.87	84.21	88.78
Drinking	34.61	28.57	31.30	22.85	28.57	25.39	38.15	85.71	52.80
Enter/Exit	89.47	82.35	85.76	89.47	100	94.44	76.00	100	86.36
**Average**	**81.31**	**79.92**	**80.40**	**77.82**	**81.08**	**79.19**	**72.85**	**73.47**	**71.52**
$\left[t^{+},t^{-}\right]=\left[2,5\right]$	**SVM**			**kNN**			**C4.5**		
**Activity**	**Pre**	**Acc**	**F1-sc**	**Pre**	**Acc**	**F1-sc**	**Pre**	**Acc**	**F1-sc**
Sleep	90.47	77.08	83.24	90.90	77.08	83.42	92.85	29.16	44.39
Toilet	66.10	71.69	68.78	67.18	86.79	75.74	82.05	64.15	72.00
Cooking	78.21	85.56	81.72	73.03	79.38	76.07	45.78	52.57	48.94
Eating	89.01	94.47	91.66	87.57	93.25	90.32	91.83	58.89	71.76
Watching TV	88.09	100	93.67	77.78	77.78	77.78	79.16	100	88.37
Phone	95.74	93.47	94.59	97.56	86.95	91.95	85.41	86.95	86.17
Dressing	92.95	94.20	93.57	93.05	95.65	94.33	75.00	62.31	68.07
Brushing Teeth	86.20	87.71	86.95	80.00	77.19	78.57	64.38	84.21	79.97
Drinking	58.33	40.00	47.45	48.14	40.00	43.69	30.00	74.28	42.73
Enter/Exit	0	0	0	71.42	64.70	67.90	69.23	47.05	56.03
**Average**	**74.51**	**74.42**	**74.46**	**78.67**	**77.87**	**78.27**	**71.57**	**65.96**	**68.65**
**Learning Time**	**SVM**			**kNN**			**C4.5**		
Average time in mobile device (in ms)	**998**			**25**			**1980**		

**Table 4:** Results with fuzzy features (spatial): precision (Pre), recall (Rec) and F1-score (F1-sc).

$\left[t^{+},t^{-}\right]=\left[0,1\right]$	**SVM**			**kNN**			**C4.5**		
**Activity**	**Pre**	**Acc**	**F1-sc**	**Pre**	**Acc**	**F1-sc**	**Pre**	**Acc**	**F1-sc**
Sleep	90.24	77.08	83.14	92.30	77.08	84.01	79.62	91.66	85.22
Toilet	84.90	86.79	85.83	89.58	84.90	87.18	88.00	92.45	90.17
Cooking	63.02	86.59	72.95	60.97	73.19	66.52	44.71	75.25	56.09
Eating	90.38	91.41	90.89	82.05	92.63	87.02	85.08	71.77	77.86
Watching TV	100	94.44	97.14	97.05	94.44	95.73	96.96	91.66	94.24
Phone	100	91.30	95.45	100	95.65	97.77	87.17	93.47	90.21
Dressing	93.15	98.55	95.77	83.75	97.10	89.93	73.21	73.91	73.56
Brushing Teeth	95.74	82.45	88.60	86.27	91.22	88.68	83.67	82.45	83.06
Drinking	50.00	71.42	58.82	44.73	62.85	52.27	32.14	77.14	45.37
Enter/Exit	100	88.23	93.75	82.35	94.11	87.84	55.55	94.11	69.86
**Average**	**86.74**	**86.83**	**86.23**	**81.90**	**86.32**	**83.69**	**72.61**	**84.39**	**76.56**
$\left[t^{+},t^{-}\right]=\left[1,3\right]$	**SVM**			**kNN**			**C4.5**		
**Activity**	**Pre**	**Acc**	**F1-sc**	**Pre**	**Acc**	**F1-sc**	**Pre**	**Acc**	**F1-sc**
Sleep	92.68	77.08	84.16	86.66	77.08	81.59	75.00	68.75	71.73
Toilet	71.66	83.01	76.92	91.11	79.24	84.76	88.09	71.69	79.05
Cooking	79.79	91.75	85.35	79.74	83.50	81.58	54.71	40.20	46.35
Eating	94.80	92.63	93.70	87.80	93.86	90.73	87.96	62.57	73.12
Watching TV	94.87	97.22	96.03	94.87	97.22	96.03	97.22	94.44	95.81
Phone	95.65	93.47	94.55	95.45	91.30	93.33	97.95	100	98.96
Dressing	89.61	100	94.52	87.65	98.55	92.78	68.25	69.56	68.90
Brushing Teeth	88.46	84.21	86.28	85.45	85.96	85.70	78.00	68.42	72.89
Drinking	57.14	62.85	59.86	58.06	60.00	59.01	24.07	80.00	37.01
Enter/Exit	90.00	100	94.73	87.50	82.35	84.84	77.27	100	87.17
**Average**	**85.46**	**88.22**	**86.61**	**85.43**	**84.90**	**85.03**	**74.85**	**75.56**	**73.10**
$\left[t^{+},t^{-}\right]=\left[2,5\right]$	**SVM**			**kNN**			**C4.5**		
**Activity**	**Pre**	**Acc**	**F1-sc**	**Pre**	**Acc**	**F1-sc**	**Pre**	**Acc**	**F1-sc**
Sleep	87.17	72.91	79.41	92.30	95.83	94.03	81.39	72.91	76.92
Toilet	63.79	69.81	66.66	77.04	90.56	83.26	66.66	75.47	70.79
Cooking	81.72	88.65	85.04	80.88	73.19	76.84	61.67	45.36	52.27
Eating	94.00	90.18	92.05	90.96	93.25	92.09	88.88	55.21	68.11
Watching TV	92.68	100	96.20	88.88	100	94.11	92.85	100	96.29
Phone	97.91	97.82	97.87	97.95	100	98.96	100	67.39	80.51
Dressing	92.75	95.65	94.18	90.27	95.65	92.88	88.09	55.07	67.77
Brushing Teeth	86.95	73.68	79.77	84.74	91.22	87.86	72.72	19.29	30.50
Drinking	64.28	80.00	71.28	59.09	74.28	65.82	28.43	82.85	42.33
Enter/Exit	81.25	94.11	87.21	88.88	94.11	91.42	80.95	88.23	84.43
**Average**	**84.25**	**86.28**	**85.25**	**85.10**	**90.81**	**87.86**	**76.16**	**66.18**	**70.82**
**Learning Time**	**SVM**			**kNN**			**C4.5**		
Average time in mobile device (in ms)	**2676**			**23**			**5382**		

**Table 5:** Results with fuzzy features (spatial and temporal): precision (Pre), recall (Rec) and F1-score (F1-sc).

$\left[t^{+},t^{-}\right]=\left[3,5\right]$	**SVM**			**kNN**			**C4.5**		
**Activity**	**Pre**	**Acc**	**F1-sc**	**Pre**	**Acc**	**F1-sc**	**Pre**	**Acc**	**F1-sc**
Sleep	90.24	77.08	83.14	92.30	77.08	84.01	79.62	91.66	85.22
Toilet	84.90	86.79	85.83	89.58	84.90	87.18	88.00	92.45	90.17
Cooking	63.02	86.59	72.95	60.97	73.19	66.52	44.71	75.25	56.09
Eating	90.38	91.41	90.89	82.05	92.63	87.02	85.08	71.77	77.86
Watching TV	100	94.44	97.14	97.05	94.44	95.73	96.96	91.66	94.24
Phone	100	91.30	95.45	100	95.65	97.77	87.17	93.47	90.21
Dressing	93.15	98.55	95.77	83.75	97.10	89.93	73.21	73.91	73.56
Brushing Teeth	95.74	82.45	88.60	86.27	91.22	88.68	83.67	82.45	83.06
Drinking	50.00	71.42	58.82	44.73	62.85	52.27	32.14	77.14	45.37
Enter/Exit	100	88.23	93.75	82.35	94.11	87.84	55.55	94.11	69.86
**Average**	**86.74**	**86.83**	**86.23**	**81.90**	**86.32**	**83.69**	**72.61**	**84.39**	**76.56**
$\left[t^{+},t^{-}\right]=\left[8,13\right]$	**SVM**			**kNN**			**C4.5**		
**Activity**	**Pre**	**Acc**	**F1-sc**	**Pre**	**Acc**	**F1-sc**	**Pre**	**Acc**	**F1-sc**
Sleep	93.02	89.58	91.27	91.83	93.75	92.78	59.25	66.66	62.74
Toilet	84.09	75.47	79.54	85.10	77.35	81.04	71.87	47.16	56.95
Cooking	94.38	91.75	93.04	94.56	93.81	94.18	72.09	40.20	51.62
Eating	97.87	86.50	91.83	90.11	93.25	91.65	83.69	58.89	69.13
Watching TV	90.00	75.00	81.81	95.23	100	97.56	91.66	55.55	69.18
Phone	97.82	95.65	96.72	92.45	100	96.07	100	100	100
Dressing	95.65	97.10	96.37	93.93	92.75	93.34	83.67	57.97	68.49
Brushing Teeth	87.23	73.68	79.88	89.28	89.47	89.37	35.96	68.42	47.14
Drinking	75.67	77.14	76.40	80.55	80.00	80.27	27.38	65.71	38.65
Enter/Exit	100	100	100	73.07	100	84.44	100	82.35	90.32
**Average**	**91.57**	**86.18**	**88.80**	**88.61**	**92.04**	**90.29**	**72.56**	**64.29**	**68.17**
**Learning Time**	**SVM**			**kNN**			**C4.5**		
Average time in mobile device (in ms)	**2128**			**24**			**4308.5**		

**Table 6:** Results with extended baseline features: precision (Pre), recall (Rec) and F1-score (F1-sc).

$\left[t^{+},t^{-}\right]=\left[0,1\right]$	**SVM**			**kNN**			**C4.5**		
**Activity**	**Pre**	**Acc**	**F1-sc**	**Pre**	**Acc**	**F1-sc**	**Pre**	**Acc**	**F1-sc**
Sleep	84.44	77.08	80.59	77.08	77.08	77.08	80.64	56.25	66.27
Toilet	78.57	62.26	69.47	67.85	79.24	73.11	93.02	86.79	89.79
Cooking	76.04	84.53	80.06	68.57	69.07	68.82	55.20	82.47	66.13
Eating	85.22	95.09	89.88	81.81	93.25	87.16	85.71	77.30	81.29
Watching TV	94.73	94.44	94.59	85.71	94.44	89.86	97.14	94.44	95.77
Phone	100	91.30	95.45	100	93.47	96.62	78.18	93.47	85.14
Dressing	92.10	100	95.89	86.58	100	92.81	86.41	100	92.71
Brushing Teeth	88.63	74.54	80.98	69.64	78.18	73.66	66.67	47.27	55.31
Drinking	74.19	76.47	75.31	61.22	94.11	74.18	43.28	91.17	58.70
Enter/Exit	84.61	88.23	86.38	84.21	94.11	88.88	80.00	94.11	86.48
**Average**	**85.85**	**84.39**	**84.86**	**78.27**	**87.29**	**82.22**	**76.62**	**82.33**	77.76
$\left[t^{+},t^{-}\right]=\left[5,3\right]$	**SVM**			**kNN**			**C4.5**		
**Activity**	**Pre**	**Acc**	**F1-sc**	**Pre**	**Acc**	**F1-sc**	**Pre**	**Acc**	**F1-sc**
Sleep	86.66	87.50	87.08	82.69	91.66	86.94	76.74	68.75	72.52
Toilet	83.78	58.49	68.88	93.61	83.01	88.00	80.48	62.26	70.21
Cooking	88.37	86.59	87.47	91.02	82.47	86.53	57.30	65.97	61.33
Eating	95.27	90.79	92.98	88.63	95.09	91.75	85.57	61.96	71.88
Watching TV	85.00	94.44	89.47	85.71	97.22	91.10	82.85	86.11	84.45
Phone	97.72	93.47	95.55	93.75	95.65	94.69	100	100	100
Dressing	94.20	98.55	96.32	85.18	98.55	91.38	94.24	94.20	93.30
Brushing Teeth	91.48	90.90	91.19	86.67	87.27	87.97	87.17	69.09	77.08
Drinking	85.71	94.11	89.71	86.48	97.05	91.46	55.31	91.17	68.85
Enter/Exit	95.00	100	97.43	79.16	100	88.37	100	100	100
**Average**	**90.32**	**89.48**	**89.90**	**87.49**	**92.80**	**90.07**	**81.78**	**79.95**	**80.86**
$\left[t^{+},t^{-}\right]=\left[15,8\right]$	**SVM**			**kNN**			**C4.5**		
**Activity**	**Pre**	**Acc**	**F1-sc**	**Pre**	**Acc**	**F1-sc**	**Pre**	**Acc**	**F1-sc**
Sleep	84.61	68.75	75.86	88.67	95.83	92.11	64.00	66.66	65.30
Toilet	92.50	71.69	80.78	90.69	75.47	82.38	84.44	77.35	80.74
Cooking	95.45	89.69	92.48	95.23	86.59	90.71	49.07	56.70	52.71
Eating	96.07	90.18	93.03	89.28	92.02	90.63	71.83	37.42	49.20
Watching TV	94.44	94.44	94.44	86.04	100	92.49	100	44.44	61.53
Phone	100	93.47	96.62	90.38	100	94.94	100	100	100
Dressing	94.52	97.10	95.79	89.85	92.75	91.28	95.23	62.31	75.33
Brushing Teeth	89.74	63.63	74.46	92.45	90.90	91.67	73.80	56.36	63.91
Drinking	97.05	100	98.50	81.17	100	93.15	76.19	94.11	84.21
Enter/Exit	100	100	100	82.60	100	90.47	100	82.35	90.32
**Average**	**94.44**	**86.89**	**90.51**	**89.24**	**93.35**	**91.25**	**81.45**	**67.74**	**73.98**

**Table 7:** S1: Non Binary (inertial + location) sensors. S2: non-location (binary + inertial), S3: non-inertial (binary + location), S4: only inertial.

$\left[t^{+},t^{-}\right]=\left[8,13\right]$	**S1**			**S2**		
**Activity**	**Pre**	**Acc**	**F1-sc**	**Pre**	**Acc**	**F1-sc**
Sleep	91.30	89.58	90.43	85.71	91.66	88.59
Toilet	76.47	66.03	70.98	90.90	77.35	83.58
Cooking	92.68	82.47	87.28	93.75	80.41	86.57
Eating	91.19	90.79	90.99	90.06	92.63	91.33
Watching TV	92.30	97.22	94.70	83.33	97.22	89.74
Phone	90.19	97.82	93.85	87.03	100	93.06
Dressing	71.01	79.71	75.11	88.00	97.10	92.32
Brushing Teeth	92.59	90.90	91.74	91.11	81.81	86.21
Drinking	72.34	100	83.95	84.61	100	91.66
Enter/Exit	73.07	100	84.44	82.60	100	90.47
**Average**	84.34	89.45	86.82	87.71	91.82	89.72
$\left[t^{+},t^{-}\right]=\left[8,13\right]$	**S3**			**S4**		
**Activity**	**Pre**	**Acc**	**F1-sc**	**Pre**	**Acc**	**F1-sc**
Sleep	79.62	85.41	82.42	66.12	89.58	76.08
Toilet	76.92	75.47	76.19	47.54	60.37	53.19
Cooking	97.77	94.84	96.28	66.66	61.85	64.17
Eating	87.57	93.86	90.60	91.91	82.20	86.78
Watching TV	82.22	97.22	89.09	50.00	52.77	51.35
Phone	96.00	95.65	95.82	87.50	91.30	89.36
Dressing	88.31	97.10	92.49	38.59	39.13	38.86
Brushing Teeth	70.00	85.45	76.95	95.45	78.18	85.95
Drinking	100	97.05	98.50	49.18	97.05	65.28
Enter/Exit	90.47	100	94.99	81.25	82.35	81.79
**Average**	86.89	92.20	89.47	67.42	73.48	70.32

## Abbreviations

The following abbreviations are used in this manuscript:

UWB	Ultra-Wide-Band
IoT	Internet of Things
BLE	Bluetooth Low Energy
MQTT	Message Queue Telemetry Transport
JSON	JavaScript Object Notation
AR	Activity Recognition
FTW	Fuzzy Temporal Window

## Appendix A. Linguistic Scale of Fuzzy Terms by Means of Triangular Membership Functions

A linguistic scale for a given environmental sensor $s^{i}$ is defined by: (i) interval values $\left[L_{1},\ldots \;L_{g}\right]$ and (ii) granularity *g*. Each term $A_{l}^{i},l\in \left[1,g\right]$ is characterized by using a triangular membership function $\mu_{\bar{A_{l}^{i}}}\left(x\right)$ [55], which is defined by the interval values $L_{i-1},L_{i},L_{i+1}$ as $\mu_{\bar{A_{l}^{i}}}\left(x\right)=TRS\left(x\right)\left[L_{i-1},L_{i},L_{i+1}\right]$, where:

(A1) $$TRS\left(x\right)\left[l_{i-1},l_{i},l_{i+1}\right]=\left\{\begin{array}{cc} 0 & x\leq l_{i-1}\\ \left(x-l_{i-1}\right)/\left(l_{i}-l_{i-1}\right) & l_{i-1}<x<l_{i}\\ \left(l_{i+1}-x\right)/\left(l_{i+1}-l_{i}\right) & l_{i}<x<l_{i+1}\\ 0 & l_{i+1}\leq x \end{array}\right.$$

## Appendix B. Aggregating Fuzzy Temporal Windows and Terms

For a given fuzzy term $V_{r}$ and a fuzzy temporal window $T_{k}$ defined over a sensor stream $S^{i}=\left\{v_{t}^{i}\right\}$, we define the aggregation $V_{r}\cup T_{k}$ in a given current time $t^{*}$ as:

(A2) $$\begin{array}{c} V_{r}\cap T_{k}\left(v_{t}^{i},t^{*}\right)=V_{r}\left(v_{t}^{i}\right)\cap T_{k}\left(\Delta t^{*}\right),\Delta t^{*}=t^{*}-t\in \left[0,1\right]\\ V_{r}^{i}\cup T_{k}^{i}\left(t^{*}\right)=V_{r}^{i}\cup T_{k}^{i}\left(S^{i},t^{*}\right)=\underset{\bar{v_{t}^{i}}\in S^{i}}{⋃}V_{r}\cap T_{k}\left(v_{t}^{i},t^{*}\right)\in \left[0,1\right] \end{array}$$

Using max-min [35] as an operation to model the t-norm and co-norm, we obtain:

(A3) $$\begin{array}{c} V_{r}^{i}\cup T_{k}^{i}\left(t^{*}\right)==\max_{t\in S^{i}}\left(min\left(V_{r}\left(v_{t}^{i}\right),T_{k}\left(\Delta t\right)\right)\right)\in \left[0,1\right] \end{array}$$

## Appendix C. Representation of Fuzzy Temporal Windows using Trapezoidal Membership Functions

Each TFW $T_{k}$ is described by a trapezoidal function based on the time interval from a previous time $t^{j}$ to the current time $t^{*}$: $T_{k}\left(\Delta t^{j}\right)\left[l_{1},l_{2},l_{3},l_{4}\right]$ and a fuzzy set characterized by a membership function whose shape corresponds to a trapezoidal function. The well-known trapezoidal membership functions are defined by a lower limit $l_{1}$, an upper limit $l_{4}$, a lower support limit $l_{2}$, and an upper support limit $l_{3}$ (refer to Equation (A4)):

(A4) $$TS\left(x\right)\left[l_{1},l_{2},l_{3},l_{4}\right]=\left\{\begin{array}{cc} 0 & x\leq l_{1}\\ \left(x-l_{1}\right)/\left(l_{2}-l_{1}\right) & l_{1}<x<l_{2}\\ 1 & l_{2}\leq x\leq l_{3}\\ \left(l_{4}-x\right)/\left(l_{4}-l_{3}\right) & l_{3}<x<l_{4}\\ 0 & l_{4}\leq x \end{array}\right.$$
